# Quantitative Wear Models for Microscale Material Removal

**DOI:** 10.3390/nano16100623

**Published:** 2026-05-18

**Authors:** Kailin Luo, Sijing Chen, Hai Li, Jian Liang, Ming Sheng, Qiuyang Tan, Yang Wang, Dingshun She, Li Zhong

**Affiliations:** 1 SEU-FEI Nano-Pico Center, Key Laboratory of MEMS of Ministry of Education, School of Integrated Circuits, Southeast University, Nanjing 210096, China; 230228381@seu.edu.cn (K.L.); 101300228@seu.edu.cn (S.C.); lihai@tyust.edu.cn (H.L.); 230238453@seu.edu.cn (J.L.); 230229329@seu.edu.cn (M.S.); 230218157@seu.edu.cn (Q.T.); 2 Research Institute of Frontier Science, Southwest Jiaotong University, Chengdu 610031, China; 3 School of Engineering and Technology, China University of Geosciences (Beijing), Beijing 100083, China

**Keywords:** microscale material removal, quantitative wear models, critical factors, prediction accuracy

## Abstract

Wear in microscale material removal is difficult to predict because material loss can proceed through several distinct pathways, including plastic deformation, adhesion, atom-by-atom attrition, tribochemical reactions, oxidation-assisted removal, and fracture. Since these mechanisms operate under different contact and environmental conditions, no single wear law is reliable across all cases. This review examines the main quantitative wear models used in microscale material removal, from classical Archard-type and Reye-type relations to atomistic Arrhenius-type descriptions and models developed for adhesive, tribochemical, oxidation-related, and fracture-dominated wear. Attention is given to the assumptions behind these models, the regimes in which they remain useful, and the conditions under which their predictions begin to fail. The discussion also considers how material properties, tool characteristics, operating conditions, and environmental factors act alone and in combination to influence wear behavior and the reliability of different models. Across the literature, a consistent conclusion is that model selection is most reliable when it is based on the active wear mechanism and the evolving contact state. On this basis, practical guidelines are outlined for different classes of contacts, and current limitations are discussed, including poor treatment of regime transitions, difficulty in parameter identification, and the gap between atomistic models and engineering use. Future progress will depend on multi-regime modeling, better treatment of coupled effects, and improved in situ characterization under realistic operating conditions.

## 1. Introduction

As demands for machining precision continue to rise in fields such as semiconductor manufacturing and ultra-precision instrumentation, traditional manufacturing techniques have become insufficient for precise manipulation at the microscale [1,2,3]. Microscale material removal techniques enable accurate material ablation spanning micron to atomic dimensions, effectively minimizing damage and preserving subsurface integrity [4,5]. This dramatically elevates process controllability and machining quality, offering critical technological support for ultra-precision machining and atomic-scale manufacturing [6,7,8,9].

Recent years have witnessed significant advancements in microscale material removal techniques, including ultra-precision diamond cutting [10,11], scanning probe-based nanofabrication, such as atomic force microscopy (AFM) [12,13,14] and scanning tunneling microscopy (STM) [15,16,17], and tribochemically assisted polishing [18,19,20]. These techniques have greatly expanded the achievable limits of surface quality and dimensional control, enabling material removal with nanometer- and even atomic-level precision. However, as the characteristic processing scale decreases, material removal behavior becomes increasingly sensitive to local contact conditions, material structure, interfacial bonding, and environmental effects [21,22,23,24]. Consequently, the underlying wear and removal mechanisms become more diverse and significantly more scale-dependent than those assumed in conventional macroscale machining [25,26]. This growing mechanistic complexity highlights the need for quantitative wear models with stronger physical grounding and improved predictive capability.

Quantitative wear models provide the theoretical basis for correlating interfacial interactions with wear volume, material removal rate, and surface evolution [27,28]. A variety of modeling approaches have been developed for this purpose, including classical phenomenological formulations such as the Archard and Reye models, as well as atomistic and mechanochemical descriptions, particularly Arrhenius-type models, that are more suitable for interpreting microscale and nanoscale removal processes [29,30,31]. These models have contributed substantially to the interpretation of specific wear phenomena and have provided useful guidance for process design under selected conditions [32,33].

Nevertheless, the current landscape of quantitative wear modeling remains far from unified. Many existing models were originally formulated for simplified or macroscale contact conditions, and their direct transfer to microscale material removal is therefore not straightforward [34,35]. In practice, microscale removal often involves multiple coexisting pathways, together with scale-dependent interfacial phenomena that can alter the relative roles of mechanical, chemical, and environmental contributions [36,37,38,39]. As a result, the applicability of different models is often condition-dependent, and their physical assumptions and predictive boundaries are not always clearly established. Although important progress has been made in individual material systems and processing scenarios, a coherent framework for comparing and evaluating these models across different removal regimes is still lacking.

Although previous studies have reviewed wear models and predictive equations from broader tribological perspectives, a systematic synthesis focused specifically on quantitative wear models for microscale material removal remains limited [40,41]. Existing reviews more commonly emphasize machining technologies [42,43,44], tribological behavior [45,46,47], or individual removal mechanisms [48,49], while comparatively less attention has been paid to critically comparing wear models in terms of their physical foundations, applicability, and limitations across different removal regimes. Such a model-centered synthesis is needed not only to organize current knowledge but also to clarify where existing models are effective, where their predictive boundaries lie, and what is required for the development of more predictive and transferable modeling frameworks.

In this context, the present review provides a critical assessment of quantitative wear models for microscale material removal, with particular focus on the Archard model, the Reye model, and atomistic wear models. It first reviews the development of representative models and critically examines their physical foundations, applicability, and limitations in interpreting material removal mechanisms. Furthermore, the critical factors influencing model accuracy are comprehensively discussed, including intrinsic material properties (anisotropy and diffusivity), processing tool properties (atomic surface configuration, chemical activity, and wear), operational parameters (sliding speed), and environmental conditions (temperature and atmosphere). Relevant evidence from atomistic, continuum, and multiscale simulations, together with available experimental studies, is considered where it helps to evaluate model applicability, limitations, and the factors governing their performance. Finally, the review presents strategies to enhance the accuracy of quantitative wear models for microscale material removal, ultimately facilitating the advancement of atomic-scale manufacturing.

## 2. Quantitative Wear Models for Microscale Material Removal

Over the past few decades, quantitative wear models for microscopic material removal have evolved from empirical macroscopic wear laws to atomistically informed descriptions of elementary removal events. These models can be broadly classified according to their physical basis and dominant range of applicability. The empirical Archard model and the energy-dissipation-based Reye model, both originally developed for macroscopic adhesive and abrasive wear, have been extended to microscale material removal, particularly when removal is dominated by plastic deformation. In contrast, atomistic wear models are intended to describe discrete atomic-scale removal events and are therefore more relevant when bond rupture, stress-assisted activation, or interfacial adhesion governs material removal. Importantly, different removal mechanisms often require different atomistic frameworks: Arrhenius-type models have been mainly applied to ionic and covalent systems, whereas emerging adhesive-wear models are more suitable for metallic contacts. In this section, these representative model families are reviewed in terms of their physical basis, applicability, and limitations, followed by a comparative assessment of their relevance across scales and removal regimes.

### 2.1. The Archard Model

As an empirical law, the Archard model has undergone a series of refinements, leading to widespread application for predicting macroscale wear [50,51]. Originally formulated by Archard in 1953, the model established a framework for adhesive wear between metallic asperities [29]. During sliding, local adhesion may occur through interfacial bonding and diffusion, and continued motion can detach material fragments upon separation of the newly formed junctions. This picture leads to the relation:

V = k_ad_ A_r_ S, (1)

where V is the wear volume, A_r_ is the real contact area, S is the sliding distance, and k_ad_ is a proportional factor representing the probability of metallic contact adhesion on rough surfaces leading to wear [52]. Consistent with contact mechanics principles, the actual contact area of contacting asperities undergoing plastic deformation is determined by Tabor’s hardness criterion [53]:

A_r_ = N/H, (2)

where N represents the applied normal load and H represents the material hardness. Rabinowicz [54] demonstrated the model’s applicability even for asperities with dissimilar mechanical properties, although the physical interpretation of k_ad_ becomes geometry-dependent on the harder asperity. This broader applicability led to a more general form of the Archard model for elastoplastic-dominated wear:

V = k N S/H,(3)

where k is the wear coefficient, influenced by material structures, surface roughness, sliding velocity, temperature, and the lubrication state of the contact region [55]. Owing to its simple form and practical utility, the model was later generalized far beyond its original metallic adhesive wear setting and has since been applied to a wide range of material systems and wear configurations [56,57,58].

Beyond macroscopic applications, the Archard model has demonstrated applicability in predicting microscale material removal processes, as evidenced by multiple studies [59,60]. For instance, Chung et al. [60] used AFM experiments to examine the validity of the model at microscale dimensions and showed that it can still provide a reasonable description of material removal when the underlying mechanism remains similar to that assumed in the macroscopic framework.

**Figure 1:**
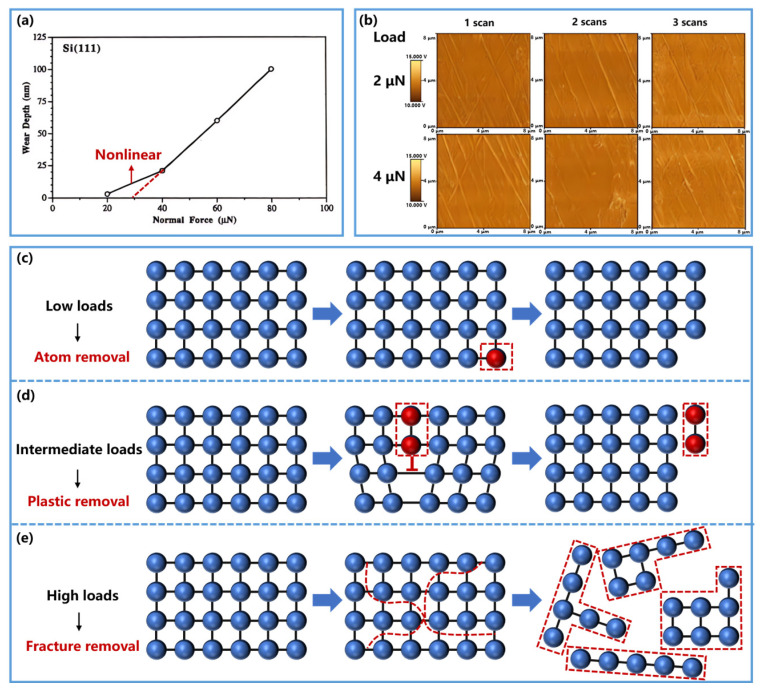
The limitations of the Archard model. (**a**) Wear depth as a function of normal force for Si (111) after one cycle. In the 20–40 μN load range, the relationship becomes nonlinear, deviating from the linear prediction of the Archard model. The red dashed line represents the linear extrapolation from the 40–80 μN range. Reproduced with permission from Ref. [61]. Copyright 1998, Elsevier. (**b**) The AFM images of the worn material region under different scans at low loads. These images show negligible wear, confirming that no obvious material removal occurs. Reproduced with permission from Ref. [62]. Copyright 2009, Elsevier. (**c**–**e**) Schematic illustration of material removal mechanisms under different loads. (**c**) At low loads, material is removed in an atom-by-atom manner. (**d**) At intermediate loads, material removal is governed by plastic regimes. (**e**) At high loads, material is removed through cleavage fracture. Blue spheres represent intact lattice atoms of the material matrix; blue arrows indicate the direction of process evolution; red spheres denote removed atoms; red dashed boxes show the extent and final morphology of lattice damage; and red dashed lines indicate the damage path of crack propagation.

However, recent studies have revealed clear limitations in its microscale applicability, particularly under extreme loading conditions [61,62,63,64,65]. Bhushan et al. [61] observed a nonlinear relationship at low applied forces, where the volume of removed material is markedly smaller than the value predicted by the linear prediction, as illustrated in Figure 1a. Colaco et al. [62] further verified that no obvious material removal occurs under low loads, as evidenced by AFM images (Figure 1b). Moreover, Walker et al. [63] attributed this deviation to a transition in the removal mechanism, showing that under low loads, material removal proceeds in an atom-by-atom manner, which lies outside the continuum assumptions of the Archard model. At the other extreme, Guo et al. [65] found that under high loads the material removal volume substantially exceeded the linear Archard prediction, with cleavage fracture identified as the dominant source of deviation. Collectively, these studies indicate that the Archard model performs best in the intermediate regime, where material removal is governed primarily by plastic deformation, as illustrated in Figure 1d, where dislocation slip dominates under moderate loads. By contrast, the model loses predictive accuracy at both ends of the regime spectrum: in the atomic-scale regime, it can overestimate removal by orders of magnitude because discrete atom detachment replaces continuum plasticity (Figure 1c), whereas in the high-load regime, it fails to capture the rapid increase in removal associated with cleavage fracture and other brittle processes (Figure 1e).

### 2.2. The Reye Model

The Reye model is an energy-based wear law that assumes the wear volume to be proportional to the frictional work dissipated during sliding [66,67]. Its general form is:

V = k ∫ F ds,(4)

where V denotes the wear volume, k is the wear coefficient, which is relative to the interface strength of the material or the tool material, F is the friction force, and s is the sliding distance. Under conditions where friction can be directly related to the energy dissipated at the contact interface, this formulation provides a convenient phenomenological description of wear. Subsequent studies by Rabinowicz and Villaggio confirmed its applicability at the macroscale [68,69].

At the microscale, the Reye model has been shown to describe material removal under conditions in which the process is dominated primarily by plastic deformation [70,71,72]. In such cases, frictional work can provide a more direct measure of the energy driving irreversible deformation and material removal than load alone. For example, AFM-based scratching experiments by Agrawal [70] identified an approximately linear relationship between wear volume and total dissipated energy under constant applied force. Consistent with this trend, long-timescale coarse-grained molecular dynamics simulations by Zhao et al. [72] showed that, for plasticity-dominated single-asperity removal, the Reye model predicts wear volume more accurately than the Archard model. These studies suggest that the Reye model is most applicable when material removal is governed predominantly by a single plasticity-controlled mechanism.

**Figure 2:**
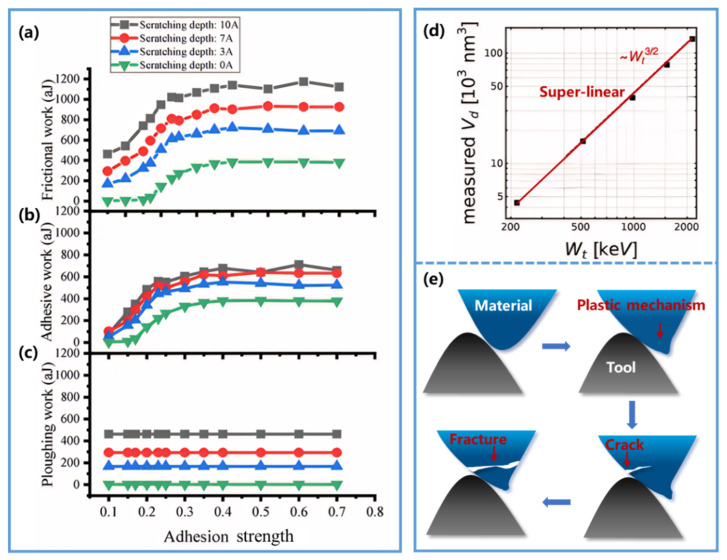
The limitations of the Reye model. (**a**–**c**) Influence of adhesive strength and removal depth (0 Å, 3 Å, 7 Å, 10 Å) on the Reye model: Relationship between (**a**) frictional work, (**b**) adhesive work, and (**c**) plowing work with adhesive strength at different removal depths. Reproduced with permission from Ref. [75]. Copyright 2022, SAGE. (**d**) The relationship between measured material removal and frictional work. Black data points represent experimentally measured removal volumes, exhibiting a superlinear relationship characterized by the red dashed line. Reprinted from Ref. [77]. (**e**) Schematic of the material removal process. Both plastic deformation and cleavage fracture occur during material removal. Blue asperity represents the material being removed; black asperity represents the removal tool; blue arrows indicate the direction of process evolution; red arrows trace the sequence of plastic deformation followed by crack initiation and eventual fracture.

However, increasing evidence indicates that this linear energy-dissipation assumption breaks down once the removal process becomes mechanistically more complex [73,74,75,76,77]. Rather than being converted into wear volume through a fixed proportionality, the dissipated frictional work at the nanoscale is partitioned among multiple channels, including defect storage, frictional heating, adhesive interactions, plowing-induced plastic deformation, and, in some cases, fracture. Liu et al. [73], combining experiments with simulations, showed that the input mechanical work during sliding can be redistributed into frictional heating, internal energy, and strain energy, and that this partitioning varies with temperature and deformation state. Their results therefore provide evidence that frictional work is not converted into material removal through a fixed, condition-independent ratio. Along the same line, Hu et al. [74] reported a nonlinear relationship between removal volume and frictional work under low adhesive-strength conditions. Joneidi et al. [75] further demonstrated that the relative contributions of adhesive work and plowing work evolve with both adhesive strength and removal depth: at shallow depths, adhesion plays a more significant role, whereas with increasing depth, the plowing contribution progressively dominates (Figure 2a–c). This evolution implies that the proportionality factor in the Reye model cannot be regarded as constant across different contact conditions. A more pronounced deviation was reported by Joaquin et al. [77], who found a superlinear relationship between measured removal volume (V_d_) and frictional work (W_t_), characterized by V_d_ ∝ W_t_^(3/2)^, as shown in Figure 2d. They attributed this behavior to the simultaneous operation of plastic deformation and cleavage fracture during material removal (Figure 2e). Under such mixed-mechanism conditions, the single-energy-pathway assumption of the Reye model is no longer valid.

Taken together, these studies indicate that the Reye model should be regarded as a condition-dependent relation at the microscale, rather than a universally applicable wear law. It should also be noted that, compared with Archard-type relations, the applicability limits of the Reye model at the microscale have been established predominantly through atomistic and coarse-grained simulations rather than through extensive experimental verification. This does not necessarily reflect a lack of relevance, but rather the experimental difficulty of simultaneously quantifying removal volume, frictional work, and their partitioning into adhesive, plowing, thermal, and fracture-related contributions during nanoscale contact. Existing AFM or nanoscratch experiments mainly support the model in plasticity-dominated regimes, whereas systematic experimental validation of its failure boundaries remains limited. This gap between mechanistic simulation and direct experimental validation remains one of the key challenges in establishing a quantitatively reliable microscale Reye-type wear law. Therefore, the current understanding of the Reye model at the microscale should be regarded as mechanism-informed and partially supported by experiments, but its quantitative applicability limits are still not fully established experimentally.

### 2.3. Atomistic Wear Models

At the atomic scale, classical wear laws such as the Archard and Reye models become increasingly inadequate because material removal is no longer governed by continuum-level load or frictional work alone, but by discrete atomic events such as bond rupture, atomic transfer, surface reconstruction, and thermally activated defect evolution. To account for these processes, atomic wear models have been developed to describe wear from the perspective of elementary removal events. As in the preceding sections, these models can be discussed in terms of their governing physical assumptions, mathematical forms, and applicability domains. Broadly, existing atomic wear models can be classified into two major categories: Arrhenius-type models, which describe wear as a thermally activated bond-breaking process, and metallic atomic wear models, which emphasize adhesive junction evolution, contact size, real contact area, and deformation-mediated material removal.

**Figure 3:**
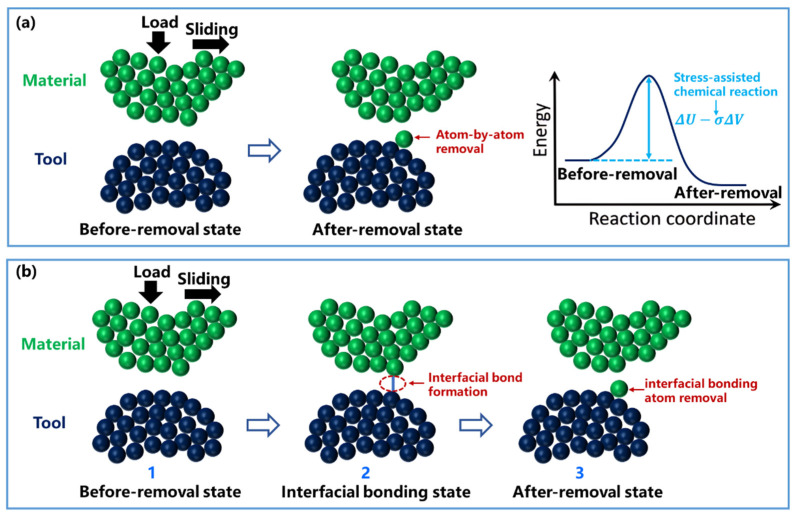
Atomic-scale material removal processes. (**a**) A stress-assisted single-step atom-by-atom removal process, where the energy barrier before and after material removal is reduced through applied stress. (**b**) A two-step atom-by-atom removal process, consisting of interfacial bond formation (1 and 2) and interfacial bonding atom removal (2 and 3). Green spheres represent the material being removed; dark blue spheres represent the removal tool; hollow dark blue arrows indicate the direction of process evolution; red arrows denote different pathways of atom removal; the red dashed box indicates the bonding state at the tool–material interface; and the blue solid line represents the formation of interfacial bonds.

Arrhenius-type atomic wear models treat atomistic wear as a thermally activated process in which the removal rate is controlled by the probability of overcoming an energy barrier [78]. A general expression can be written as:

k = k_0_ exp (−(∆G_act_)/(k_B_T)), (5)

where k is the atomic wear rate, k_0_ is the effective attempt frequency, ∆G_act_ is the effective activation energy, k_B_ is the Boltzmann constant, and T is the absolute temperature. Building on this framework, Sheehan et al. [79], Gotsmann et al. [80], and Jacobs et al. [81] showed experimentally that applied stress can reduce the effective activation energy during sliding (Figure 3a). Based on this stress-assisted reaction picture, atom-by-atom removal was described as a single-step interfacial chemical process, and the atomic loss rate, k_atom-loss_ (atoms removed per second), was expressed as:

k_atom-loss_ = k_0_ exp (−(ΔU − σΔV)/(k_B_T)), (6)

where ΔU is the stress-free activation internal energy, σ is the stress component reducing the energy barrier, and ΔV is the activation volume. More recently, Wang et al. [82] proposed a refined two-step mechanism in which atom-by-atom removal involves both interfacial bond formation (states 1 and 2 in Figure 3b) and subsequent removal of interfacial bonding atoms (states 2 and 3 in Figure 3b). Consequently, the model was described as:

N_ib_ = N_rc_ exp (−(ΔU_ib_ − W_σ_)/(k_B_T)), (7)

N_wear_ = N_ib_ exp (−(ΔU_wear_ − W_τ_)/(k_B_T)) t, (8)

where N_rc_ is the number of all atoms in the real contact surface, N_ib_ is the number of surface atoms participating in interfacial chemical bonds, N_wear_ is the number of worn atoms, t is the time of the material removal process, ΔU_ib_ is the activation energy of interfacial bond formation, ΔU_wear_ is the removal of interfacial bonding atoms, W_σ_ and W_τ_ are the external work per atom induced by normal stress and shear stress, respectively. Compared with the single-step formulation, this two-step model explicitly distinguishes bond formation from bond-breaking-assisted removal, thereby providing a more detailed description of tribochemical atomistic wear.

Despite their wide use, Arrhenius-type models still face several unresolved issues. One concerns the choice of the stress parameter used to reduce the activation barrier: Sheehan et al. [79] and Yang et al. [83] emphasized shear stress, whereas Jacobs et al. [81] and Park et al. [84] used average contact stress. Another issue is the limited physical transparency and transferability of the fitted activation parameters, which are often treated as effective quantities rather than directly measurable material constants [31,82]. For example, although diamond-like carbon (DLC) and silicon differ substantially in bonding character, the fitted activation energies reported for DLC (0.98 eV) and silicon (1.0 eV) are remarkably similar [82]. More fundamentally, these models have been developed and validated primarily for materials dominated by covalent or ionic bonding, whereas their applicability to metallic and two-dimensional material systems remains largely unexplored. Therefore, Arrhenius-type atomic wear models are most suitable for systems in which atomistic wear is controlled mainly by stress-assisted bond rupture or tribochemical activation, but their broader transferability across material classes remains uncertain.

In contrast to Arrhenius-type models, atomic wear models for metallic materials do not follow a single unified form. Instead, they are commonly developed based on adhesive junction formation, real contact area, plastic deformation, and wear-fragment generation at sliding interfaces [85,86,87,88]. Atomistic simulations by Aghababaei et al. [89] showed that the junction size of contacting asperities controls the transition in the adhesive wear mechanism: below a critical length scale, material removal proceeds mainly through atom-by-atom attrition, whereas above it, wear debris formation becomes continuum-like, Archard-type wear in metallic contacts. Consistent experimental evidence was later reported by Walker et al. [90], who observed a transition between atomic wear and elastoplastic plowing wear in nanoscale metallic contacts, with the transition depending strongly on load and contact size. Together, these studies indicate that in metallic systems, the applicability boundary between atomistic wear and Archard-like wear is largely governed by contact-size-dependent deformation mechanisms.

A second line of development has focused on extending the Archard framework to metallic wear across multiple scales [33,91,92]. For example, Lin et al. [33] introduced scale- and microstructure-dependent correction terms into the classical Archard model for nanotwinned metals, thereby linking wear behavior across macro-, micro-, and atomic-scale contacts. Such studies suggest that Archard-type relations can still be useful for metals when atomistic and microstructural effects are explicitly incorporated. A third class of metallic atomic wear models is based directly on the real contact area. Using atomistic simulations of self-mated Au asperity contact and separation, recent studies [93] have shown that the real contact area is the dominant factor controlling nanoscale adhesive wear and proposed a geometric model for estimating wear-fragment volume from the contact area. Compared with Arrhenius-type formulations, such models are more directly connected to interfacial geometry and junction evolution in metallic contacts.

In practical engineering, however, the wear behavior of metals is rarely governed by a single factor. Experimental studies on tool steels and other metallic systems have shown that wear depends jointly on microstructural features, hardness, surface roughness, applied load, and the competition between adhesive and abrasive mechanisms [94,95]. This complexity makes it difficult for purely mechanism-based models to provide universally reliable predictions. As a result, data-driven and machine-learning-assisted approaches have been increasingly applied to metallic wear prediction, incorporating multiple coupled descriptors to improve predictive capability for microscale and nanoscale wear [96,97]. Although such approaches may sacrifice some mechanistic transparency, they offer an effective route for capturing the high-dimensional parameter dependence commonly observed in engineering metallic systems.

Taken together, these studies show that metallic atomic wear is governed less by a single thermally activated bond-breaking pathway than by the competition among contact size, real contact area, adhesion, plasticity, and microstructural evolution. Existing models can therefore be grouped into contact-size-controlled transition models, Archard-type corrected models, real-contact-area-based adhesive models, and data-driven predictive models. Their coexistence reflects both the richness of metallic wear mechanisms and the fact that a universally applicable atomic wear law for metals has not yet been established.

### 2.4. Mechanism Transition, Predictive Capability, and Applicability of Wear Models

The wear models reviewed above are built on different physical descriptions of material removal and therefore correspond to different contact regimes. Archard-type models describe wear in terms of continuum-scale material loss controlled mainly by load, sliding distance, and hardness, and are most closely associated with plasticity-dominated asperity removal. Reye-type models interpret wear from the perspective of frictional energy dissipation and are applicable when material loss remains closely correlated with frictional work. Atomic-scale models, however, are more heterogeneous. For covalent or ceramic systems, wear is often described by Arrhenius-type formulations in which atom-by-atom removal is treated as a thermally activated and stress-assisted bond-breaking process. In metallic systems, by contrast, no single unified atomic wear law has been established. Instead, existing models are commonly developed from different but related perspectives, including contact-size-controlled mechanism transition, adhesion and real contact area, plastic deformation and debris generation, corrected Archard-type relations, and, more recently, data-driven descriptions that incorporate multiple coupled descriptors. Therefore, the differences among Archard-, Reye-, and atomic-scale models arise not only from scale, but also from the specific removal mechanisms and contact descriptors they are intended to represent.

At present, no single universal criterion can quantitatively distinguish these three regimes for all materials and contact conditions. Existing studies instead suggest that the dominant mechanism is jointly controlled by several related factors, most notably characteristic contact size, debris size, normal load or local contact stress, penetration depth, and interfacial adhesion. Foundational work established a critical contact size for the transition from atomic removal to plasticity-dominated wear [89], and subsequent studies further showed that fracture-dominated wear emerges in the supercritical regime and may exhibit superlinear friction-work scaling [77]. At a more local level, both debris size relative to contact size and fragment atom count have been used to differentiate atomic attrition, plasticity-assisted removal, and fracture-dominated severe wear [72,98]. In addition, the transition from atom-by-atom removal to elastoplastic plowing has been shown to occur above critical load or pressure levels, with stronger adhesion lowering the corresponding threshold [63,64,99]. Similar behavior has been reported in scratching configurations, where increasing penetration depth promotes the sequence from elastic response to plastic deformation and eventually fracture, although the transition depth itself depends strongly on adhesion conditions [65,76]. Taken together, these results indicate that wear-mechanism transitions are not governed by a single universal parameter, but by the coupled effects of contact size, stress state, penetration, and interfacial bonding, all of which reflect the same underlying competition among atomic attrition, plastic flow, and fracture-assisted debris formation. These quantities should therefore be regarded as practical mechanism-selection indicators rather than universal laws.

A related question is whether the predictive accuracy of Archard-, Reye-, and atomic-scale wear models can be quantitatively compared. In our view, such a comparison is only meaningful in a limited sense. These model families were developed for different scales and different dominant mechanisms, and they often predict different physical quantities. Archard-type models are mainly intended for continuum-scale wear volume or wear rate under plasticity-dominated conditions [50,51]. Reye-type models relate wear to dissipated frictional work [70,71]. Atomic-scale models are more often used to describe removal rate, transition threshold, or atomistic debris formation when bond rupture and local adhesion dominate [79,80,81,82,83,84,89,99]. Therefore, the predictive performance reported for each model should be interpreted within its own mechanism regime and validation context, rather than as a directly comparable universal measure across all model families.

Even so, a broad-scale dependence in model suitability can still be identified. At the macroscale, and often also at the microscale when continuum plasticity dominates, Archard-type models remain effective because contact can still be represented by averaged load- and hardness-controlled removal [100,101]. In simplified contacts in which material loss scales mainly with frictional dissipation, Reye-type models can also provide useful predictions [70,71,72]. As the characteristic contact size decreases toward the nanoscale, however, local adhesion, bond rupture, atomic transfer, and discrete debris nucleation increasingly control the wear response, so that atomic-scale models become more physically appropriate [102,103]. Conversely, once loading, penetration, or adhesion becomes sufficiently large for fracture and large debris formation to emerge, the assumptions of simple linear wear laws become less reliable, and piecewise, corrected, or hybrid descriptions are generally required. Accordingly, the evolution from macro to micro to nano should be understood primarily as a shift in dominant mechanism and model validity, rather than as a universally quantifiable change in numerical prediction accuracy.

Another important limitation is that practical wear rarely proceeds through a single isolated mechanism. In real tribological contacts, atomic attrition, metallic adhesion and transfer, plastic deformation, fracture, thermal activation, and sometimes tribochemical effects may coexist and evolve during sliding [98,104,105]. For this reason, no single model can be expected to remain accurate across the full transition from atomic-scale removal to plasticity-dominated wear and then to fracture-controlled severe wear. Existing approaches usually address this difficulty by introducing effective coefficients, transition thresholds, corrected constitutive relations, or hybrid multi-regime frameworks [89,99,106]. Accordingly, the most meaningful comparison among wear models is not a simple ranking by nominal “accuracy”, but a mechanism-informed comparison of their physical basis, applicable regime, dominant removal pathway, predictive characteristics, and major limitations. Following this principle, Table 1 summarizes the Archard, Reye, and atomic-scale wear models from these perspectives and provides a practical guide for model selection under different contact conditions.

## 3. Critical Factors Affecting the Accuracy of Quantitative Wear Models

In practical microscale and nanoscale material-removal processes, the accuracy of quantitative wear models is affected not only by the mathematical form of the model itself, but also by whether the selected descriptors adequately capture the actual removal mechanism. Besides intrinsic material properties, model performance can be strongly influenced by processing tool characteristics, operational parameters, and environmental conditions. These factors become increasingly important at small scales, where anisotropy, diffusion, atomic-scale contact structure, and interfacial reactions are no longer averaged out. As a result, changes in one or more of these factors may shift the dominant removal mode among plasticity, atom-by-atom attrition, tribochemical wear, adhesion-mediated transfer, and fracture, thereby limiting the predictive accuracy of single-mechanism models. This section therefore discusses the critical factors affecting model accuracy from the perspectives of material properties, tool properties, operational parameters, environmental conditions, and their coupled effects.

### 3.1. Intrinsic Material Properties

Intrinsic material properties affect quantitative wear models primarily by changing the local deformation pathway and the kinetic response of interfacial material removal. Among these properties, crystallographic anisotropy and atomic diffusivity are especially important at microscale and nanoscale contacts, where the involved material volume is too limited to average out local structural effects.

Crystallographic anisotropy is inherent to crystalline materials, but it is rarely included explicitly in existing wear models. In macroscopic removal processes, the contact usually spans many grains with different orientations, and orientation-dependent behavior is effectively homogenized. In microscale removal, however, the number of grains involved is limited, and in some cases, the contact may interact with only one grain or even one crystallographic surface. Under such conditions, anisotropy can directly influence whether the material undergoes dislocation slip, phase transformation, amorphization, or fracture, and can therefore determine which wear model is physically appropriate [107,108,109,110,111]. For example, He et al. [107] showed that in nanoscale single-crystal Si, plastic deformation under <100> loading is dominated by dislocation slip, whereas <111> loading leads to phase-transformation-mediated amorphization, demonstrating a strong orientation dependence of deformation mechanisms. Likewise, Sainath et al. [108] reported anisotropic deformation behavior in body-centered cubic (BCC) Fe nanowires, where the <100> orientation favors deformation twinning while the <111> orientation primarily exhibits dislocation slip. These results indicate that orientation is not merely a secondary descriptor of deformation; rather, it can switch the dominant removal mechanism itself.

**Figure 4:**
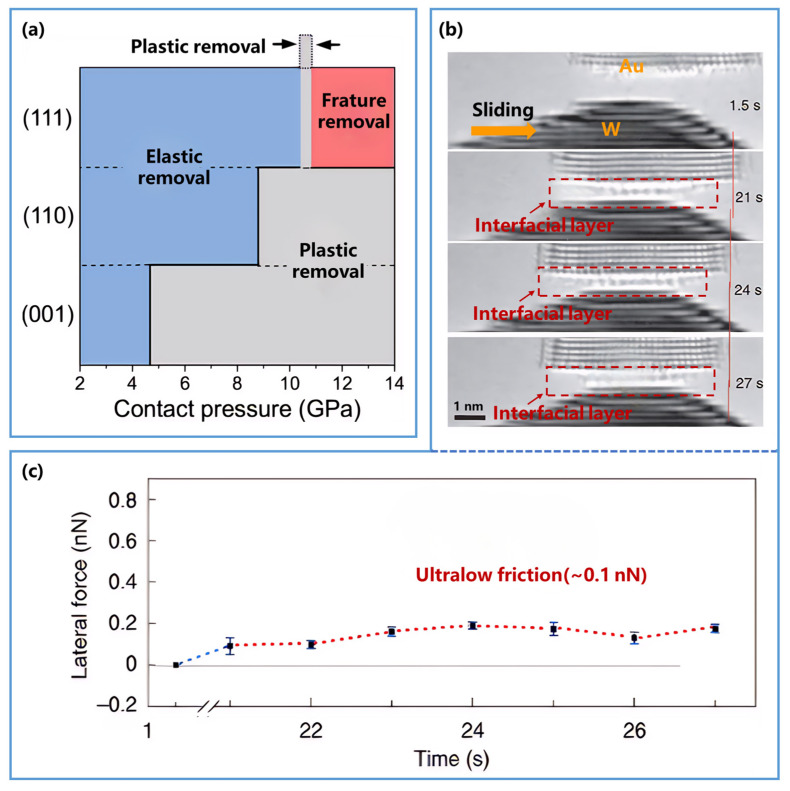
Influence of material intrinsic properties on material removal behavior. (**a**) Schematic illustration of material removal mechanisms for different orientations of CaF_2_ as a function of contact pressure. The (001) and (110) surfaces exhibit purely elastic-plastic removal across all contact pressures, whereas the (111) orientation undergoes a transition to fracture-dominated removal at elevated pressures. Reproduced with permission from Ref. [110]. Copyright 2017, Elsevier. (**b**) High-resolution transmission electron microscopy (HRTEM) images of W and Au asperities during the sliding process. Orange arrows indicate the sliding direction; the red dashed box highlights the interfacial layer structure. (**c**) Time-resolved lateral force measurements. During sliding contact, Au atomic diffusion generates a loosely packed interfacial layer, which mediates ultralow friction. This provides direct evidence that diffusion-mediated interfacial energy modification critically influences material removal behavior. Reproduced with permission from Ref. [116]. Copyright 2022, Springer Nature.

The direct consequence for wear modeling was further demonstrated by Chen et al. [110], who showed that different crystal orientations of CaF_2_ exhibit distinct material-removal mechanisms with increasing contact pressure. As shown in Figure 4a, the (001) and (110) surfaces remain within an elastic–plastic removal regime over the investigated pressure range, so their wear behavior can still be reasonably captured by Archard-type scaling. By contrast, the (111) surface undergoes a transition to fracture-dominated removal at higher contact pressures, causing clear deviation from Archard-model predictions. This comparison has important quantitative implications: the same nominal pressure range does not correspond to the same governing mechanism across different orientations, meaning that the fitting coefficient or even the model form itself may no longer be transferable from one crystallographic surface to another. Thus, anisotropy should be regarded as a mechanism-selection variable rather than only a material constant.

Another intrinsic factor of growing importance at small scales is atomic diffusivity, especially in metallic systems [112,113,114,115,116,117,118]. Unlike bulk-scale behavior, nanoscale metallic contacts can exhibit substantial diffusion-driven restructuring during sliding or indentation. Sun et al. [112] demonstrated that Ag nanoparticles undergo diffusion-assisted geometric evolution, which dynamically changes the real contact area and therefore alters the stress field assumed in simplified wear relations. Zhong et al. [115] further showed that diffusion-mediated plasticity can substantially modify deformation behavior, indicating that hardness- or stress-based descriptions alone may become insufficient. Most notably, He et al. [116] identified the formation of a loosely packed interfacial layer induced by atomic diffusion at a metallic contact (Figure 4b). This diffusion-mediated interfacial restructuring was accompanied by ultralow friction (Figure 4c): the average lateral force decreased to ~0.1 nN under the tested conditions, compared with ~0.8 nN for typical metal–metal contacts without such a layer, corresponding to nearly an order-of-magnitude reduction. Such diffusion-driven interfacial evolution implies that the effective contact state, interfacial shear response, and local material properties are not constant during sliding, which challenges wear models based on fixed contact geometry or time-invariant material parameters.

Overall, intrinsic material properties influence model accuracy mainly through two routes: first, by controlling the dominant deformation or removal mechanism; and second, by modifying the local kinetic and structural state of the contact. This explains why model transferability across materials, orientations, and scales often remains limited even when nominal loading conditions appear similar.

### 3.2. Processing Tool Properties

The processing tool affects model accuracy not only through its nominal geometry, but also through its atomic-scale surface configuration, chemical activity, and progressive wear state. These effects become increasingly important at small scales, where the tool–material interface can no longer be idealized as a geometrically smooth and chemically inert contact.

**Figure 5:**
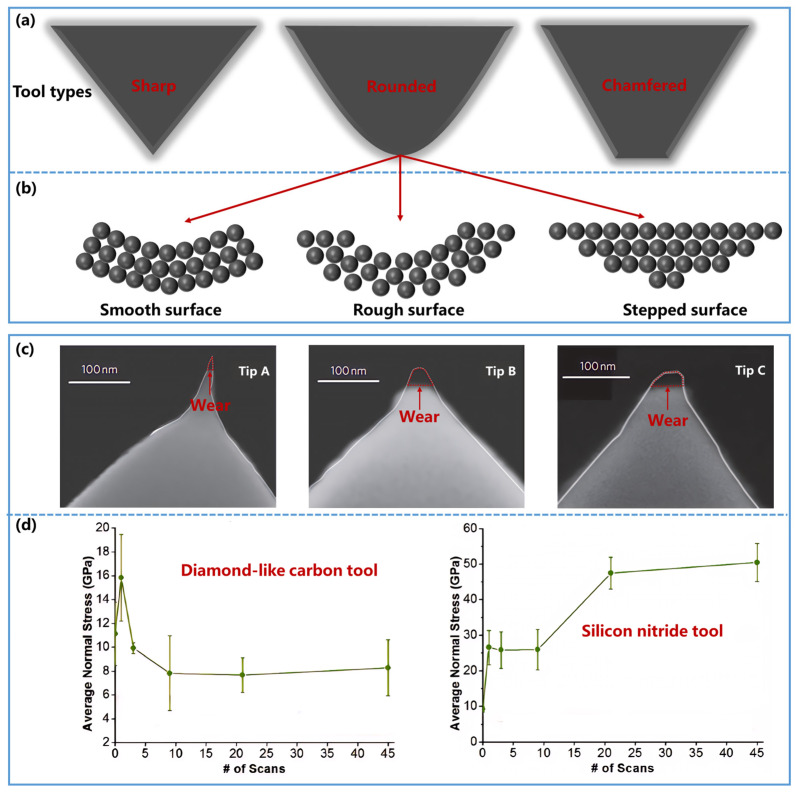
Impact of processing tool properties on quantitative models. (**a**) Classification of processing tools in macroscopic material removal processes. Tools are categorized into sharp, rounded, and chamfered types based on edge geometry. (**b**) Different atomic surface configurations of the same rounded edge geometry: These include smooth, rough, and stepped surfaces. (**c**) SEM images of wear on diamond-like carbon tool tips with varying geometries (Tip A < Tip B < Tip C in curvature radius) exhibit distinct wear deterioration during material removal, where red dashed boxes denote fractured segments at the apex. Reproduced with permission from Ref. [125]. Copyright 2010, Springer Nature. (**d**) Variation in average normal stress with increasing scan numbers for diamond-like carbon and silicon nitride tools. The symbol “#” in the horizontal axis stands for “number”. Both tool types show increased wear with higher scan counts, leading to altered average normal stress and, consequently, affecting model accuracy. Reproduced with permission from Ref. [126], Copyright 2013, American Chemical Society.

In conventional macroscale machining, tool geometry is often classified into sharp, rounded, and chamfered forms (Figure 5a), and extensive studies have shown that edge geometry strongly influences stress concentration, chip formation, and plowing behavior [119,120,121,122]. However, recent microscale investigations reveal that atomic-level surface characteristics introduce significant deviations from macroscopic behavior. Even with identical nominal geometries, tools exhibit fundamentally different interfacial interactions depending on their atomic surface configurations, which compromises the accuracy of quantitative wear models. Luan and Robbins et al. [40] employed MD simulations to analyze rounded tools with varying atomic surface characteristics, including smooth, rough, and stepped configurations (Figure 5b). Their results showed that atomic roughness and surface steps significantly modify the real contact area and local stress distribution, leading to substantial deviation from predictions based only on nominal shape. This finding indicates that, at small scales, real contact topology rather than nominal edge geometry may be the more relevant descriptor for quantitative wear modeling.

The chemical activity of the tool is another key factor because it can change the dominant material-removal mechanism and therefore the appropriate model class. As discussed in Section 2, Archard- and Reye-type models are more suitable for plasticity-dominated removal, whereas Arrhenius-type models are more relevant when atom-by-atom removal is controlled by stress-assisted bond rupture or tribochemical activation. The tool material can shift the system between these regimes. For example, highly chemically inert diamond tools primarily induce removal through plastic deformation, making the Archard model appropriate [123]. In contrast, chemically active SiO_2_ tools can facilitate interfacial bond formation and rupture, thereby favoring atomistic or tribochemical removal mechanisms better captured by Arrhenius-type models [124]. Therefore, tool chemical activity is not only a material property of the tool itself, but also a mechanism-governing factor that determines model suitability.

Tool wear further complicates quantitative prediction because the tool state evolves during processing. As shown in Figure 5c, Bhaskaran et al. [125] observed that diamond-like carbon tool tips invariably undergo wear during material removal processes, irrespective of tip geometry variations. Vahdat et al. [126] further demonstrated that such wear in different tool types can alter the average normal stress with increasing scans (Figure 5d), leading to an accumulated deviation from model predictions based on the initial tool condition. In practical terms, this means that model parameters calibrated at the beginning of processing may not remain valid as the tool degrades. Tool wear can therefore introduce a hidden time dependence into nominally steady-state wear relations. Protective coatings, such as solid lubricating layers, are often used to mitigate this problem and stabilize the interface [127,128,129], but from the modeling perspective, the more fundamental need is to account for evolving tool shape, chemistry, and contact stiffness when these changes become significant.

In summary, processing tool properties affect model accuracy by modifying the actual contact state in ways that are often neglected in simplified wear formulations. Their influence becomes particularly important when nominal geometry, constant chemical state, or non-evolving tool conditions are assumed.

### 3.3. Operational Parameters

Among operational parameters, applied load and sliding speed are the most direct variables controlling contact stress, contact duration, and energy input, and therefore strongly influence the accuracy of quantitative wear models. Although most existing models explicitly include load, the role of sliding speed is often treated only indirectly or neglected altogether.

**Figure 6:**
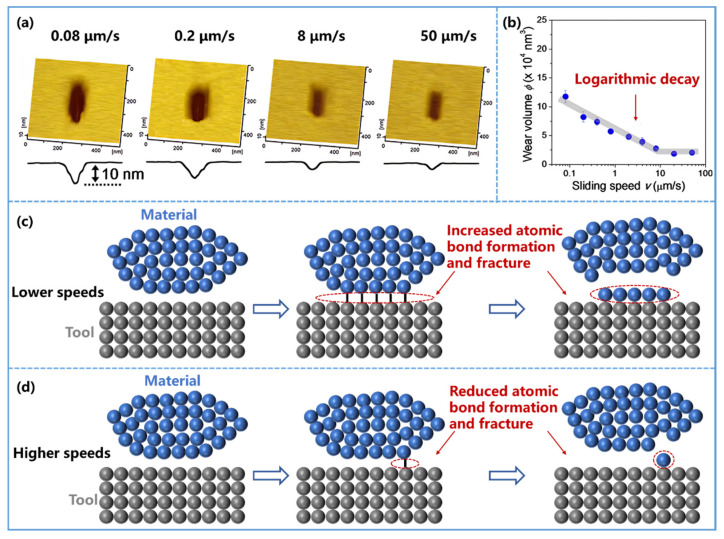
Effect of sliding speed on material removal behavior. (**a**) AFM images and corresponding cross-sectional profiles of silicon surface removal at different sliding speeds (0.08 μm/s, 0.2 μm/s, 8 μm/s, and 50 μm/s). (**b**) Removal volume of silicon surface as a function of sliding speed, showing a logarithmic decay relationship. Reprinted from Ref. [131]. (**c**) Schematic of material removal at lower sliding speeds. Increased atomic bond formation and fracture occur under these conditions, facilitating enhanced material removal. (**d**) Schematic of material removal at higher sliding speeds. Reduced atomic bond formation and fracture lead to diminished material removal. Blue spheres represent the material being removed; grey spheres represent the removal tool; hollow blue arrows indicate the direction of process evolution; black solid lines denote atoms involved in interfacial bonding; and the red dashed box indicates the region and final morphology affected by atom removal.

At small scales, sliding speed can affect wear because it changes the duration over which local stress and interfacial bonding act on the same contact region. Yu et al. [130] observed that material removal occurs more readily at lower sliding speeds. Chen et al. [131] further verified through AFM experiments that wear volume decreases with increasing sliding speed (Figure 6a) and approximately follows a logarithmic decay trend over the tested range (Figure 6b). This behavior is generally attributed to the longer contact time available at lower speed, which promotes interfacial bond formation and bond rupture, thereby facilitating material removal (Figure 6c). At higher speed, the reduced interaction time suppresses such bond-breaking events and lowers removal efficiency (Figure 6d). These observations suggest that in atomistic or tribochemical removal regimes, sliding speed affects wear through time-dependent activation kinetics rather than by load alone.

However, the influence of sliding speed is not universal. Shao et al. [99] reported that under sufficiently high contact stress, material removal becomes nearly insensitive to sliding speed. In that regime, the strong local stress promotes removal through a mechanism that can be reasonably approximated by stress- or plasticity-controlled wear, making Archard-type behavior more relevant. This contrast highlights an important point: the influence of sliding speed depends on the dominant wear mechanism and on its coupling with load. In low- to moderate-stress regimes, wear may exhibit clear kinetic sensitivity to contact time and thus deviate from load-based continuum models; in high-stress regimes, the same system may revert to stress-dominated behavior and show weaker rate sensitivity.

Beyond load and speed, contact-path kinematics can also affect wear evolution by changing the spatial accumulation of stress and damage. Kohutiar et al. [132] reported that, under identical nominal load and speed, replacing linear motion with reciprocating elliptical motion in dry sliding against 30CrNiMo8 steel increased wear by 147%, indicating that motion geometry can modify the oxidation and damage state of the contact. This suggests that path-dependent kinematics should also be considered when evaluating the applicability of quantitative wear models.

Therefore, the effect of operational parameters is better understood in terms of mechanism transitions and contact-history dependence rather than dependence on isolated variables. Quantitatively, the wear response may be characterized not only by the monotonic decrease in wear volume with sliding speed, but also by the fitted logarithmic dependence where applicable, the characteristic stress threshold above which speed sensitivity becomes weak, and the influence of contact-path kinematics on cumulative damage. Such descriptors help clarify the regime dependence of model validity and explain why a single quantitative wear model may fail when load, speed, and motion path jointly shift the dominant removal mechanism.

### 3.4. Environmental Conditions

Environmental conditions influence wear-model accuracy by modifying interfacial reaction pathways, activation barriers, oxidation behavior, adsorption states, thermal activation, and mass transport at the contact interface. These effects are particularly important in microscale and nanoscale wear, where the removal process is often governed not only by mechanical stress but also by chemically assisted bond rupture, surface passivation, or thermally activated material transfer. As a result, environmental variables may alter both the wear rate and the dominant wear mechanism, thereby limiting the transferability of models calibrated under a single atmosphere or temperature.

**Figure 7:**
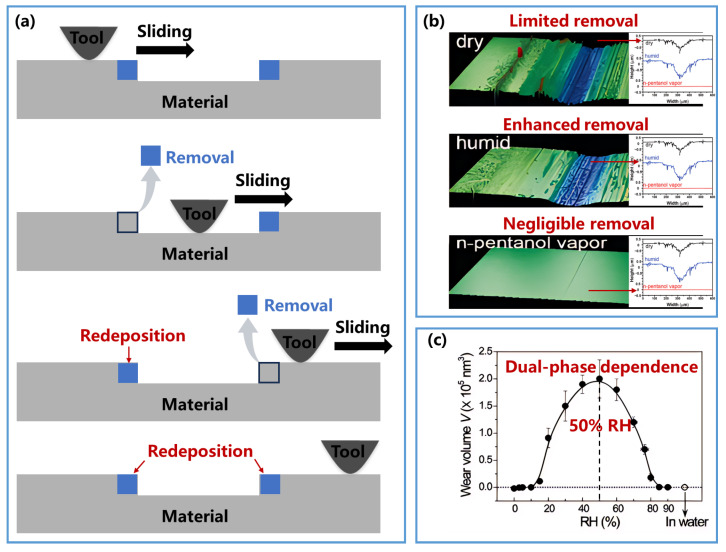
Influence of environmental conditions on material removal behavior. (**a**) Schematic of material removal at elevated temperatures. High-temperature-induced diffusion leads to material redeposition, thereby reducing removal volume and causing deviation from Arrhenius model predictions. Light grey regular shapes represent the material being removed; dark grey asperities represent the removal tool; the black arrow indicates the sliding direction; the blue box indicates removed material that subsequently redeposits; light grey arrows denote removal; red arrows denote redeposition. (**b**) Optical profilometry images and characteristic line profiles of SiO_2_ removal under dry conditions, 50% relative humidity (RH), and 50% n-pentanol vapor pressure with an applied load of 0.7 N. Removal volume is limited in dry environments, significantly enhanced at 50% RH, and nearly negligible under 50% n-pentanol vapor pressure. Reproduced with permission from Ref. [134]. Copyright 2009, American Chemical Society. (**c**) Removal volume of SiO_2_ as a function of RH exhibits a dual-phase dependence: below 50% RH, removal volume increases with humidity, while above this threshold, it decreases. Reproduced with permission from Ref. [135]. Copyright 2015, American Chemical Society.

Temperature is one of the most fundamental environmental variables. In atomistic and tribochemical wear models, it is commonly introduced through an Arrhenius-type activation term, according to which increasing temperature lowers the effective activation barrier and accelerates material removal. Liu et al. [73] confirmed this temperature dependence through both simulations and experiments. However, temperature does not always increase net wear monotonically. Wang et al. [133] reported that material removal volume can decrease at elevated temperature, in apparent contradiction to a simple Arrhenius prediction. This anomaly was attributed to thermally activated redeposition, in which enhanced diffusion promotes reattachment of removed species to the surface (Figure 7a). The implication is that temperature can accelerate both forward removal and reverse recovery processes. As a result, a single-barrier Arrhenius model may become insufficient when multiple competing thermally activated pathways coexist.

The surrounding atmosphere also plays a major role by changing interfacial adsorption and bond-formation conditions. Barnette et al. [134] compared SiO_2_ removal under dry conditions, 50% relative humidity (RH), and 50% n-pentanol vapor pressure. As shown in Figure 7b, wear is limited in dry air, enhanced in humid air, and strongly suppressed in n-pentanol vapor. The underlying reason is that adsorbed water reduces the effective barrier for interfacial bond rupture, whereas n-pentanol adsorption inhibits the interfacial reactions needed for material removal. Wang et al. [135] further showed that SiO_2_ removal exhibits a two-stage humidity dependence (Figure 7c): below about 50% RH, removal increases with humidity, while above this level it decreases. This reversal was attributed to the formation of a thick, solid-like water layer that hinders direct interfacial bond formation and fracture. Thus, the effect of humidity is not simply monotonic; instead, it involves a threshold-like transition between reaction-promoting and contact-screening regimes.

Oxidation is another critical environmental factor, particularly for metallic materials operating at elevated temperatures or in reactive atmospheres, because it can alter surface composition, adhesion tendency, and the dominant wear mode. In general, when a stable and continuous oxide layer is formed, direct metal–metal adhesion is reduced, and wear may shift from adhesive or severe metallic wear to mild oxidative wear with a lower wear rate. For example, Ilo et al. [136] developed an oxidation-coupled Archard-type framework to describe the transition from adhesive wear to mild oxidative wear. Similarly, Dong et al. [137] showed that an oxidized surface suppresses adhesive wear and enhances wettability, thereby improving wear resistance. However, the protective role of oxidation is not universal. Cui et al. [138] found that for Cr–Mo–V cast steels tested in air at 400 °C that oxidation can either reduce wear through the formation of protective oxide films or lead to severe wear when the oxide layer becomes unstable and spalls, with the final outcome depending strongly on substrate microstructure and film failure mode. More generally, Lim et al. [139] demonstrated, through wear mechanism maps for steels, that oxidation determines both the onset of mild oxidative wear and the transition between mild and severe wear regimes. Collectively, these studies indicate that oxidation is not merely a background chemical effect, but rather a dynamic state variable governing transitions among adhesive wear, mild oxidative wear, and severe oxidative wear [140].

Overall, environmental conditions influence model accuracy by altering not only the wear rate but also the governing wear mechanism itself. Temperature can introduce competing thermally activated processes; humidity and surrounding vapor species can alter adsorption and tribochemical reaction pathways, and oxidation can drive transitions among distinct wear regimes depending on oxide-film stability. These effects are often nonlinear and may involve threshold behavior or mechanism switching, which cannot be adequately represented by a single constant fitting parameter. Consequently, quantitative wear models calibrated under one environmental condition may lose predictive accuracy when transferred to another unless such environmentally induced changes in mechanism are explicitly incorporated.

### 3.5. Coupled Effects Among Multiple Factors

In practical tribological systems, wear is seldom controlled by a single factor acting independently. Instead, it emerges from the interplay of material properties, operating conditions, and environmental conditions. This is one of the main reasons why quantitative wear models calibrated under simplified conditions often lose accuracy when applied to practical systems.

One common form of coupling is that between mechanical loading and oxidation. Tewari et al. [141] developed an analytical model for oxidative wear by combining chemical kinetics with Hertzian contact mechanics, showing that wear volume depends on both load and sliding speed when removal is governed by a stable oxide layer. Milan et al. [142] similarly reported in high-temperature sliding of multicomponent Fe alloys that the effects of temperature and normal load are strongly mediated by oxide formation and compacted debris layers, which reduce both friction and wear. In such systems, load and speed do not act as purely mechanical variables; their influence is shaped by the chemical evolution of the interface.

At small scales, the coupling between stress, time, and environment becomes even more evident because wear is often governed by tribochemical bond formation. Xiao et al. [124] showed that for Si/SiO_2_ nanocontacts, water significantly lowers the threshold pressure for wear, while repeated sliding further reduces this threshold through energy accumulation in the contact region. Tian et al. [143] further found that for silica nanocontacts, frictional aging increases approximately linearly with both the normal load and the logarithm of the hold time, linking load dependence directly to time-dependent interfacial bond formation. These findings show that in tribochemical wear, load, humidity, and contact time act jointly through stress-assisted reaction kinetics rather than purely mechanical deformation.

Coupling can also involve geometry, adhesion, temperature, and external fields. Zhong et al. [144] showed, using atomistic simulations of adhesive wear between rough Al asperities, that stronger adhesion, greater geometric overlap, and higher initial temperature all increase wear, reflecting the combined roles of interface strength, thermal activation, and deformation state. In a more complex engineering context, Lei et al. [145] demonstrated that for current-carrying metallic contacts, increasing electrical input shifts the wear mechanism from primarily mechanical removal to electro-thermo-mechanical wear through Joule heating, frictional heating, and local softening or melting. These examples highlight that when multiple driving fields act simultaneously, wear behavior can no longer be described reliably by purely mechanical models.

Overall, the challenge for quantitative wear modeling is not simply to include more variables, but to capture how those variables interact. Load can alter oxidation kinetics, speed can change the time available for tribochemical activation, humidity can shift the stress threshold for bond rupture, and thermal or electrical inputs can open entirely different removal pathways. Future progress will therefore depend on models that treat wear as a coupled, evolving process rather than the sum of independent factors.

## 4. Conclusions and Prospects

Microscale material removal is central to ultra-precision manufacturing, yet its quantitative description remains difficult because wear at this scale does not arise from a single process. Material may be removed by plastic deformation, adhesion and transfer, atom-by-atom attrition, tribochemical reaction, oxidation-assisted wear or fracture, depending on the material system and the contact conditions. These pathways are affected in different ways by load, contact size, sliding speed, temperature, humidity, interfacial chemistry, and electrical input. As a result, a model that performs well in one regime may become unreliable when the dominant removal pathway changes.

The literature considered in this review points to a consistent pattern. Archard-type and Reye-type relations remain useful when contact conditions are relatively stable, and wear is governed mainly by mild plastic deformation or can be treated in an averaged energetic sense. When removal proceeds atom by atom, especially in covalent or ionic materials under small loads and at very small contacts, atomistic Arrhenius-type descriptions are generally more appropriate because they link wear directly to stress-assisted bond breaking. Metallic contacts are often more difficult to describe using a simple law. Where adhesion, junction growth, local plasticity, and material transfer are strongly coupled, predictive performance depends much more on how the evolving contact state is represented. Under humid, oxidizing, tribochemical, high-temperature, or current-carrying conditions, the interface may change continuously during sliding, and the wear coefficient can no longer be treated as a fixed material constant.

A central point that emerges from these studies is that model selection should be guided first by the active removal mechanism and the conditions under which it operates. In practice, models developed for one set of conditions are often used for different materials or environments without sufficient consideration of whether the underlying assumptions still hold. Many of the differences reported in the literature are likely to arise for this reason. The most useful question is therefore not which wear equation is most familiar, but whether its assumptions remain valid for the contact being analyzed.

This has direct implications for applications. In ultra-precision polishing [146], AFM-based nanomachining [147,148], and related micro- and nanoscale machining processes [149], removal is often controlled by atomic attrition or tribochemical effects, while surface integrity and subsurface damage must be carefully managed. In such cases, mechanism-sensitive and environment-dependent models are more useful than conventional macroscopic wear laws. In microelectromechanical systems and precision sliding interfaces, repeated contact at small scales can lead to adhesion, contact aging, and humidity-dependent degradation that are difficult to capture using models developed for larger, more stable interfaces. In metallic tribosystems operating at elevated temperature or under electrical current, oxidation, Joule heating, and interfacial softening may alter the wear pathway and limit the usefulness of models calibrated under ambient mechanical conditions. In these practical settings, quantitative wear modeling is important not only for estimating material loss but also for choosing process windows, comparing operating conditions, and improving long-term reliability.

At the same time, the limits of current models remain clear. Many are built around a single dominant mechanism and therefore have difficulty describing transitions from one wear mode to another. Parameters that matter most in activation-based or environmentally coupled descriptions, such as the true contact state, local bonding condition, reaction activity, and subsurface structural evolution, are often difficult to access directly. There is also a persistent scale gap. Physically detailed models are often not easy to implement in process design, whereas simpler engineering expressions may lose the mechanism-level information that actually controls wear at small scales. These issues continue to limit predictive accuracy, especially when operating conditions differ from those used for model calibration.

To improve predictive capability and broaden applicability, future work should focus on the following three directions:

(1) Developing unified multi-regime wear models: A realistic unified model should not force all wear behavior into a single fixed equation. A more realistic approach is to treat microscale wear as a process that can shift between different mechanisms as the interface evolves. This will require models that can connect descriptions of atomic attrition, plastic deformation, adhesion, tribochemical wear, oxidation-assisted wear, and fracture within a common framework, while also identifying the conditions under which transitions occur. Stress level, contact size, temperature, interfacial chemistry, and surface evolution are likely to be among the most important descriptors for this purpose. A model built on such variables would offer a more credible route towards predictive modeling across a wider range of materials and operating conditions.
(2) Modeling coupled effects in microscale wear: At microscale contacts, wear is rarely governed by mechanics alone. Thermal effects, humidity, surface reactions, oxidation, and electrical input often act together and reshape the interface as sliding proceeds. Models that treat these factors as independent corrections are unlikely to remain reliable over a broad range of conditions. Future progress will require closer integration of mechanics with heat transfer, interfacial chemistry, oxidation kinetics, and electrical effects where relevant. Data-driven methods may also prove useful, especially for parameter identification, regime recognition, and efficient approximation of complex nonlinear responses. At the continuum level, Archard- or friction-energy type wear laws can be implemented in FEM as wear-evolution or surface-update relations, while multiscale frameworks can use atomistic results to inform transition criteria, interfacial constitutive behavior, and mechanism-dependent parameters. They are likely to be most useful when combined with physically grounded modeling rather than used as a substitute for it.
(3) Advancing in situ characterization for model validation and parameter identification: Many of the quantities that control wear at small scales are still inferred indirectly after the event. This makes it difficult to test mechanistic assumptions or to assign model parameters with confidence. Better in situ characterization under realistic operating conditions will therefore be essential. High-resolution probe-based techniques, electron-microscopy-based observations, and environment-controlled experimental platforms can help track contact evolution, bond change, reaction progress, and subsurface response during removal. Such measurements are important not only for validating existing models, but also for building new ones with a stronger physical basis.

In summary, the literature offers little support for a single wear equation that can be applied reliably across all forms of microscale material removal. A more useful view is that each model is tied to a particular removal mechanism and to the contact conditions under which that mechanism dominates. Quantitative wear modeling should therefore begin with identifying the active wear process and the range over which a given description remains valid. Further progress is likely to come not from repeated adjustments of fixed empirical laws, but from models that can track changes in interface state and capture transitions between removal pathways under realistic conditions.

## Figures and Tables

**Table 1:** Comparison of quantitative wear models for microscale material removal.

Model	Principle	Dominant Mechanism	Applicable Scale	Typical Predictive Trend	Main Limitations
Archard model	V = k N S/H	Plastic deformation/adhesion	Macro to micro (plastic regime)	Reliable in plasticity-dominated wear	Fails for atom-by-atom attrition and severe fracture; k not constant across mechanisms
Reye model	V = k ∫ F ds	Energy dissipation (plasticity-dominated)	Macro to micro (single-mechanism regime)	Linear with frictional work in mild plastic wear	Limited under multi-mechanism wear; energy partition not unique
Atomic-scale Arrhenius-type model	k = k_0_ exp (−(∆G_act_)/(k_B_T))	Atom-by-atom bond rupture	Nanoscale (especially covalent/ionic systems)	Captures stress-/temperature-dependent atomic attrition	Activation parameters difficult to extract from experiments; limited for metallic and 2D materials
Atomic-scale metallic model	No single unified form	Adhesion, plasticity, transfer, debris nucleation	Nano to micro (metallic contacts)	Captures the transition from atomic wear to continuum-like wear	No unified equation; strong material, geometry, and mechanism dependence

## Data Availability

No new data were created or analyzed in this study. Data sharing is not applicable to this article.
